# The reproductive strategy in a *Chloris virgata* population in response to precipitation regimes

**DOI:** 10.1098/rsos.180607

**Published:** 2018-08-01

**Authors:** Wang Ying, Wang Chunxia, Zhang Jukui, Wang Chunqing

**Affiliations:** Key Laboratory of Songliao Aquatic Environment, Ministry of Education, Jilin Jianzhu University, Changchun 130118, People's Republic of China

**Keywords:** allometry, reproduction, seed, spike, strategy

## Abstract

Resource availability influences plant growth and reproduction. Here, a controlled experiment was conducted in order to evaluate the adaptation response of *Chloris virgata* to different precipitation conditions, and to further predict the reproductive strategy in a population of *C. virgata* under different precipitation regimes. Three regimes (low, typical and high) of water addition were used to simulate current precipitation patterns. In total 20 individuals for each treatment were analysed to compare tiller number, spike traits, seed traits, the relationship between seed size and seed number, and so on. In addition, the effects of different precipitation regimes on offspring vigour of *C. virgata* were also studied. Results indicated that tiller number, spike number, seed yield and seed number were unchanged under different water addition regimes, while seed size was about 0.5 mg at typical and high precipitation levels and was higher than that in the low precipitation level. The higher seed mass per spike and spike mass both occurred at typical and high precipitation levels. Significant positive correlations between seed mass and non-seed mass in *C. virgata* in response to precipitation regimes were largely allometric (size dependent), as was a significant negative correlation between seed size and seed number at low precipitation. The highest germination rates and seedling weights both occurred at typical and high precipitation levels. These findings showed that different precipitation regimes affected reproductive strategy of *C. virgata. Chloris virgata* will not benefit from low precipitation, while typical and high precipitation will improve seed traits and offspring vigour of this species.

## 1. Introduction

Reproduction is one of the most fundamental traits of plants. Reproductive pattern is a core component of a plant's life-history strategy and is the result of natural selection. Plants can exhibit various strategies to cope with different selection pressures for a variable available resource [1]. The growth and development of reproductive organs is affected by environmental conditions, so reproductive strategy plays an important role in plant adaptation by maximizing the overall fitness of plants growing in different environments [2].

Trade-off is one of the fundamental features of ecological strategies in plants [3]. A trade-off between growth and reproduction exists because increased allocation to one function, as the result of natural selection to cope with different growing environments, often comes at a cost to other functions [4]. Studies of trade-off between growth and reproduction are, therefore, useful in understanding the adaptation of plants to their natural environments. Variable resource allocation also affects seed traits, which are important components of seed yield [5]. In turn, seed traits, such as seed size, can affect offspring characters, so that maternal nutrient status, for example, can influence the competitive ability of offspring, including traits such as seed germination and seedling establishment [6].

Over the past several decades, China has experienced some devastating changes in precipitation, impacting on water reserves and plant production patterns [7]. Grassland degradation, resulting, in part, from drought, has been extensive on the Songnen Plain in northern China. *Chloris virgata* Sw (feather finger grass) is an annual grass of the family Poaceae, characterized by high protein content, which is widely distributed across a range of soil types. The species is tolerant of drought conditions and is the first plant to appear and form a dominant community on bare patches of alkaline soil [8]. *Chloris virgata* is, therefore, a valuable species for promising grass species in salinity–alkalinity and drought-stressed environments [9]. The precipitation regime in the region is the major factor affecting the reproductive ecology. The objective of this study was to evaluate the reproductive strategy of this important species when subjected to a simulation of the precipitation regime prevailing in the Songnen grassland, as well as lower and higher precipitation rates. The findings will inform rational regulatory and management policy in relation to water resources to maintain the health of the various ecosystems that make up northeast China.

## 2. Material and methods

### 2.1. Experimental design

The experiment was carried out at Northeast Normal University, Jilin Province, China in 2015. *Chloris virgata* seeds were collected from the Ecological Research Station for Grassland Farming, Chinese Academy of Sciences, in Changling, China (44°33 N, 123°31 E) in 2014. Twenty-four PVC pipes, 25 cm diameter and 50 cm height, were filled with local nutrient-poor sandy aeolian soil on 1 June 2015. The pipes were buried in the soil. The top edge of each pipe and the soil surface were at the same regime and 20 seeds were sown in each pipe. Ten days after sowing, the plants were thinned to five seedlings per pipe, selecting uniform and robust seedlings for retention. There were three treatments, corresponding to 120 (L: low), 200 (T: typical) and 280 (H: high) mm precipitation, which were created by adding water every 5 d to the pipes from 30 d after seedling emergence to simulate the different precipitation regimes. The typical precipitation regime was based on the region's amount and frequency of precipitation (June–October) during the period of vegetative growth averaged over the past 21 years in the semi-arid temperate steppe of Jilin province, where *C. virgata* is widely distributed. The precipitation regimes measured in wet or dry years were 40% higher or lower, respectively, than the average baseline on the data from the Songnen grassland over the past 21 years. Initially, there were eight replicate pipes for each regime. Four replicates (20 individuals per treatment) were used to determine spike and seed traits, while the other four replicates were used to collect seed and to determine seedling vigour. The final number of pipes was four for each precipitation level, giving 20 individuals. Pipes were arranged outdoors in a replicated randomized block design under a rain shelter made from transparent 0.2 mm polyethylene sheeting on 1 June 2015 (see [10]).

### 2.2. Sampling procedure and data collection

On 20 September 2015, when all *C. virgata* individuals were fully mature, the plants from four replicate pipes of each treatment were harvested at soil level. The number of reproductive tiller and non-reproductive tiller of *C. virgata* was recorded for each individual in each pipe. One spike on a first-order tiller (one first-order tiller produces one spike) was selected randomly from each plant, and the spike was separated into seeds and reproductive support tissue. Each plant was then separated into spike and non-spike parts, the number of spikes was recorded for each individual plant in each pipe and all the spikes from each plant were put into individual paper bags. In the laboratory, the non-spike parts were dried at 65°C for 48 h and then weighed. The numbers of mature seeds and unripe seeds were counted to obtain ripeness data. The seeds, unripe seeds and reproductive support tissue of each spike were dried at 65°C for 48 h in the laboratory and then weighed to obtain spike seed mass and spike mass. Fifty seeds were selected randomly from each plant, and dried at 65°C for 48 h and weighed to obtain seed size. The remaining seeds from each plant were dried at 65°C for 48 h and weighed to determine seed mass. The seed number of each plant was approximated by dividing the seed mass by the mean seed size. The seed yield then was calculated by seed mass per plant and area of the pot. Seed mass: mass of seed produced by a plant; non-seed mas: total above-ground mass excluding seed mass.

### 2.3. Germination experiment

Seeds were harvested from the plants in four replicate pipes at maturity, with all the seeds from each pipe being pooled and kept separate from those from the other replicates. Then the seeds were air-dried in the laboratory and stored until germination tests were undertaken. To determine percentage germination and germination rate, 50 seeds were selected at random from each replicate. Samples, each containing 50 seeds, were placed on moist blotting paper in 90 mm Petri dishes in October 2015. The Petri dishes were then placed in growth chambers and maintained at 20°C to 30°C with a 12 h photoperiod for 10 d. Germination was said to have occurred when the radicle had penetrated the seed coat. The number of seeds germinated was counted every day to determine percentage germination and germination rate. On the last day, 10 seedlings were selected from each replicate, and the seed coat was removed, if attached, before harvesting. The seedlings were dried at 65°C for 48 h to obtain seedling dry weight.

Germination rate = 2* *Σ* (Gi/Di), where Gi is the number of normal seedlings germinated in the *i*th day; Di is the number of days until the *i*th reading [11].

### 2.4. Data analysis

One-way ANOVA was used to analyse variables such as spike number, seed number, ripeness, spike mass, seed mass per spike, seed size, seed yield, percentage germination, germination rate and seedling weight. Unless stated to the contrary, variables were approximated to normality with homogeneous variances, permitting the use of parametric analyses. Multiple comparisons between different precipitation regimes were detected using the least significant difference test, with a critical significance level of *p *≤ 0.05. To analyse reproductive patterns allometrically and to make variances homogeneous, biomass values were log-transformed. Allometric relationships among seed mass versus non-seed mass and seed size versus seed number were analysed by the log-transformed version of the classical ‘allometric’ model: log *Y *= *a* log *X *+ *b*, where *a* is the allometric slope and *b* (intercept) is the log of the allometric coefficient. Standardized major axis (SMA) regression was used to determine the slopes and intercepts using the software package SMATR (standardized major axis tests and routines, see [12]). The slopes were first tested to determine whether they were significantly different from 1 for each allometric relationship. Differences in allometric slopes between precipitation regimes were then analysed for significance.

## 3. Results

### 3.1. Reproductive characters

**Figure 1.**
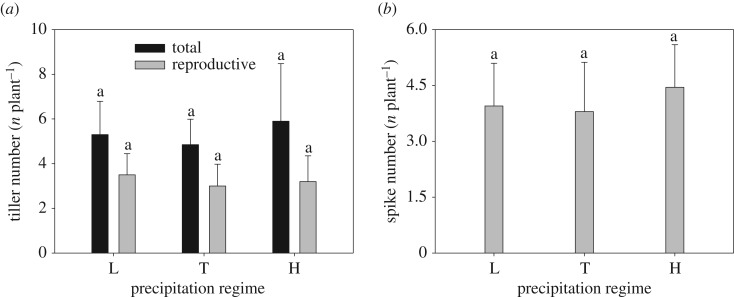
Effect of different precipitation regimes on (*a*) tiller number and (*b*) spike number (means ± 1 s.d.) of *C. virgata*. Means with the same letter are not significantly different at *p *= 0.05. L, T and H indicated low, typical and high precipitation levels, respectively (the same below).

**Figure 2.**
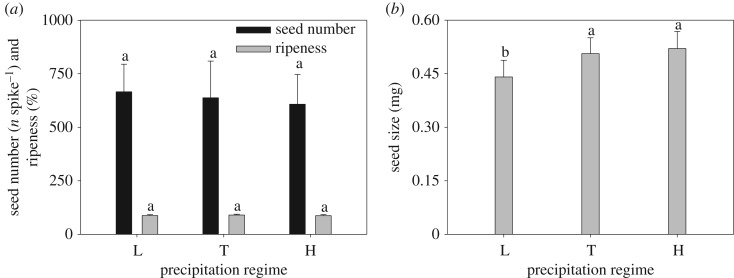
Effect of different precipitation regimes on (*a*) seed number and ripeness, and (*b*) seed size (means ± 1 s.d.) of *C. virgata*. Means with the same letter are not significantly different at *p *= 0.05.

**Figure 3.**
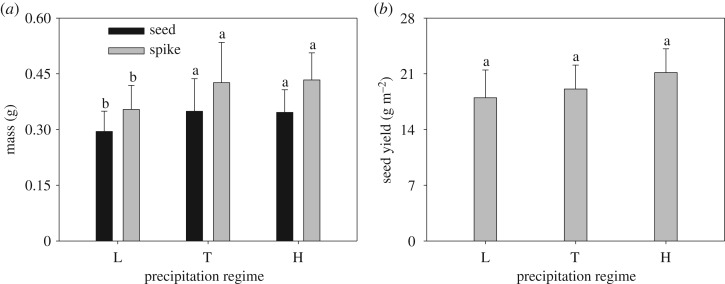
Effect of different precipitation regimes on (*a*) spike and seed mass per spike (means ± 1 s.d.), and (*b*) seed yield (means ± 1 s.d.) of *C. virgata*. Means with the same letter are not significantly different at *p *= 0.05.

The number of total tiller and reproductive tiller per plant exhibited similar trends and no significant changes were observed among different levels of precipitation treatments. The mean reproductive tiller was about 3.2 (figure 1*a*). Spike number per plant responded to precipitation regimes and remained unchanged among three precipitation regimes (figure 1*b*). Seed number and ripeness did not vary significantly with increasing precipitation (figure 2*a*). However, seed size increased significantly from low to typical precipitation, but there was no significant difference in seed size between typical- and high-precipitation regimes (figure 2*b*). Both spike mass and seed mass per spike increased from low to typical precipitation, above which there was no further significant change (figure 3*a*), while seed yield did not change significantly with increasing precipitation (figure 3*b*).

### 3.2. Reproductive allometric relationships

**Figure 4.**
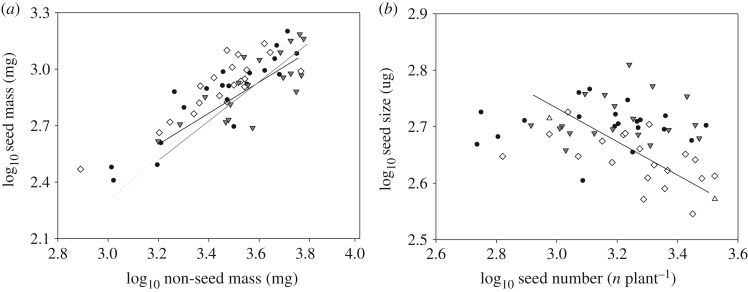
Allometric relationships of (*a*) seed mass versus non-seed mass and (*b*) seed size versus seed number at L (diamonds), T (filled circles) and H (filled inverted triangles) precipitation regimes.

The allometric relationships between seed mass and non-seed mass, and between seed size and seed number differed between the treatments (figure 4 and table 1). The slopes of the SMA regression between seed mass and non-seed mass were not significantly different between the three precipitation regimes. Significant positive correlations between log seed mass and log non-seed mass were found in all treatments (figure 4*a* and table 1). In none of the precipitation regimes were the slopes of the relationship between seed mass and non-seed mass significantly greater than 1. By contrast, the fixed negative allometric relationship between seed size and seed number at low precipitation shifted to a non-allometric relationship under typical and high precipitation (figure 4*b*). The slope of the relationship between seed size and seed number was significantly greater than −1 (lower and upper confidence intervals −0.40 and −0.17, respectively; table 1) at low precipitation.

**Table 1.** Effects of precipitation intensity on the slopes and intercepts of allometric relationships for seed mass versus non-seed mass, seed size versus seed number of *C. virgata*, using standardized major axis on the allometric model log_10_*Y* = slope × log_10_ *X* + intercept. (Values with different letters are significantly different at *p *< 0.05.)

	precipitation regime		
log_10_ *Y* versus log_10_ *X*	low	typical	high
seed mass (mg) versus non-seed mass (mg)			
slope	0.85 ^a^	1.00 ^a^	1.03 ^a^
intercept	−0.04	−0.61	−0.76
*R*^2^	0.75	0.82	0.59
CI of slope	0.66–1.10	0.82–1.24	0.75–1.42
*p*	0.00	0.00	0.00
seed size (µg) versus seed number (*n* plant^−1^)			
slope	−0.26	0.17	0.24
intercept	3.49	2.17	1.94
*R*^2^	0.25	0.00	0.02
CI of slope	−0.40–0.17	0.10–0.27	0.15–0.40
*p*	0.03	0.94	0.60

### 3.3. Seed traits

Different seed traits of *C. virgata* responded differently to the various precipitation regimes (table 2). Although percentage germination was not affected by precipitation regimes, germination rate varied significantly owing to water supply, with the slowest germination rate observed in the low precipitation pipes (table 2). Seedling weight responded similarly to germination rate (table 2).

**Table 2.** Percentage germination, germination rate and seedling weight (means ± 1 s.d.) of *C. virgata* under different precipitation regimes. (Within columns, means followed by the same letter are not significantly different at *p *= 0.05.)

precipitation regime	percentage germination (%)	germination rate	seedling weight (mg)
low	86 ± 4.3^a^	27 ± 1.7^b^	0.30 ± 0.009^b^
typical	93 ± 6.6^a^	32 ± 0.9^a^	0.35 ± 0.015^a^
high	94 ± 4.3^a^	31 ± 0.9^a^	0.39 ± 0.019^a^

## 4. Discussion

### 4.1. Reproductive characters

In arid and semi-arid regions, precipitation is one of the major factors that affects the growth and development of plants. Therefore, plant reproductive strategy in response to precipitation regime was investigated here to predict the effects of current environmental changes on species dynamics, coexistence and abundance in grasslands.

Plants will select different strategies under different biotic and abiotic environments to maximize their fitness. At first, the plants must accumulate resources for growth and then build up the reproductive machinery. Reproductive tiller number was important for yield component of *C. virgata* and our results showed that there were no significant changes in total and reproductive tiller number under different precipitation levels. The results were not consistent with those of the former works because the changes of the tiller number depended on different growth stages and different water stress levels [13–15]. Furthermore, a bud developing into a reproductive tiller or non-reproductive tiller is based on the environment and the evolutionary history of the population.

The results indicated clearly that decreasing precipitation from typical to low precipitation levels resulted in lower spike mass, seed mass and seed size, examples of the negative effects of drought on plant growth and reproduction [16]. These results were consistent with those from previous studies [17,18]. Young *et al.* [19] reported that seed number per spike and seed size were the most important components for seed yield, while Wang *et al.* [5] reported that spike number was a more important component than seed number per spike and seed size for *Leymus chinensis* seed yield.

However, seed yields were unchanged between the different precipitation regimes in this study (figure 3*b*). The results were not consistent with those from earlier studies [20,21]. The reason for this discrepancy might be that spike number included the spike number of first-order and second-order tillers in this study. While the mean spike number on first-order tillers (3.5 per plant) in the low-precipitation regime was higher than that under typical and high precipitation (2.7 and 3.3 per plant, respectively), there were no significant difference in mean spike number per plant between the three precipitation treatments. Moreover, the secondary spike was very small and produced few seeds owing to its late development in the season (data not shown). Therefore, first-order tillers were the most important components for seed yield.

### 4.2. Reproductive allometric relationships

The trade-offs between reproductive mass and non-reproductive mass, and between seed size and seed number are important factors for understanding the underlying mechanisms of species adaptation and vegetation recovery. In this study, significant positive correlations between seed mass and non-seed mass were found in all treatments. The slopes of seed mass versus non-seed mass were not significantly different from 1, which suggested that the increase in seed mass was proportional to the increase in non-seed mass. However, a significant negative correlation between seed size and seed number was found under the low precipitation treatment. The regression coefficient of log seed size versus seed number was significantly greater than −1, indicating that a marginal increase in seed size caused a proportionally smaller decrease in seed number.

*Chloris virgata* alters its water resource allocation to reproduction to improve its fitness. This indicates that precipitation is one of the vital factors that affects this species' growth and reproduction. Moreover, the seed size/seed number trade-off results from water limitation and conceals the differences in available water resources between individuals. It is reasonable to consider that different plants adapt to produce more but smaller seeds or fewer but larger seeds to improve their fitness, which increases plant competitive ability and community structure [22].

However, there were no allometric relationships under typical and high precipitation, though the seed size–seed number trade-off is a common assumption in plant reproduction ecology. This observation may be owing to the typical precipitation regime providing adequate water resource. In contrast to the individuals under the low precipitation regime, individuals at higher precipitation rates will not take up and use more of the water resource to produce larger or more flowers and seeds.

### 4.3. Seed traits

Seed morphology and physiology traits, such as seed size and seedling vigour, are vital for plant growth and development in variable environments. In this study, seed size was lower under a low-precipitation regime than under typical or high precipitation. The result was similar to that reported by Wulff [23] where seed size in *Desmodium paniculatum* L. was reduced significantly by limited water supply. Busso & Perryman [24] also confirmed that the precipitation regime, to which the mother plant is exposed, can have a significant effect on the progeny. In their study, seed size of Wyoming sagebrush (*Artemisia tridentata* ssp. *wyomingensis* Beetle and Young) in a low-precipitation year was lower than that in a high-precipitation year. In general, drought stress during early seed development decreases the seed sink potential by reducing the number of endosperm cells and amyloplasts formed, which ultimately decreases the seed size [25]. However, in the current study, seed size under the high-precipitation regime was not the highest among the three treatments. The reason might be that the soil water content under the typical precipitation regime was sufficient to support the optimal growth and development of *C. virgata*.

Additionally, seed size is an important criterion for determining seedling vigour and establishment [6,26]. In the present study, lighter seed was associated with lower germination ability and seedling establishment. In general, heavier seed results in increased germination rate, seedling survival and enhanced seedling growth, providing an advantage in terms of competitive ability when facing environmental hazards or competition with other seedlings [27,28]. Seed quality and seed yield are important factors in the establishment of new grassland and in the renewal of degraded grassland [29]. Therefore, renewal of the Songnen grassland will be impeded in dry years.

## 5. Conclusion

In conclusion, increased precipitation did not increase seed yield or seed quality in this study, which suggests that reproduction of *C. virgata* will not benefit from above-average precipitation regimes during wet years in this area in the future. It is likely, however, that the increasing frequency of dry years as a result of climate change will have a negative effect on the regeneration of *C. virgata* in arid and semi-arid areas such as the Songnen grassland*.* These results will also provide some important information on management measures for the establishment and recovery of *C. virgata* in degraded grassland.

## Data accessibility

Data deposited in Dryad, see http://dx.doi.org/10.5061/dryad.76fk344 [30].
